# Supplementary material for: Diagnosis of Malnutrition According to GLIM Criteria Predicts Complications and 6-Month Survival in Cancer Outpatients
Source: Biomedicines. 2022 Sep 6;10(9):2201. doi: 10.3390/biomedicines10092201 (PMC9496397; doi:10.3390/biomedicines10092201)
Supplement: Supplementary file 1 [file biomedicines-10-02201-s001.zip › biomedicines-1875946-supplementary.pdf]

Supplemental Table S1. Odds ratios for malnutrition-associated complications according to tumor location.

Complication	Head & Neck n=19	Colorectal n=82	Upper GI n=64
Emergency Room admission	0.61 [0.06;4.71]	2.17 [0.82;6.01]	1.92 [0.70;5.40]
Hospitalization	3.26 [0.46;33.7]	4.17 [1.48;12.2]	3.50 [1.18;11.7]
Severe infection	3.47 [0.36;112]	5.03 [1.54;17.7]	1.88 [0.57;6.98]
Mild infection	3.47 [0.36;112]	1.58 [0.58;4.28]	1.08 [0.34;3.55]
Poor pain control	0.29 [0.01;2.78]	3.39 [1.23;9.62]	5.51 [1.85;18.5]
Increase in opioid dosage	2.71 [0.41;21.5]	4.17 [1.48;12.2]	4.87 [1.58;17.6]
Toxicity	5.09 [0.46;172]	2.81 [0.45;74.6]	3.36 [0.94;14.3]
Gastrointestinal toxicity	1.90 [0.29;14.5]	1.01 [0.38;2.82]	3.54 [1.27;10.5]
Hematological toxicity		1.14 [0.31;3.66]	2.71 [0.96;8.19]
Neurological toxicity		1.00 [0.38;2.64]	1.53 [0.53;4.66]
Decrease or discontinuation of treatment	21.4 [2.14;747]	1.85 [0.58;7.34]	2.68 [0.92;8.28]
Tumor Progression	1.05 [0.14;7.54]	1.71 [0.62;4.67]	2.31 [0.79;7.26]
6-month survival	0.91 [0.08;7.94]	0.17 [0.03;0.74]	0.70 [0.22;2.14]

Supplemental Table S1. Odds ratios for malnutrition-associated complications according to tumor location.

Complication	Head & Neck n=19	Colorectal n=82	Upper GI n=64

Odds ratio and 95% confidence intervals
